# Contribution of FOS in neutrophils to venous thromboembolism via miR‐144 based on bioinformatic prediction and validation

**DOI:** 10.1111/jcmm.18370

**Published:** 2024-05-31

**Authors:** Ping Wang, Lin Zheng, Xiaotong Qi, Heng Wang, Ruijing Zhang, Liying Song, Ruihan Chen, Sheng Yan, Wenkai Chang, Jie Hu, Yuwen Wang, Haijiang Jin, Yongbin Shi, Zhihui Wu, Wenbo Zhao, Peilu Shi, Qinqin Tian, Miao Xing, Honglin Dong

**Affiliations:** ^1^ Department of Vascular Surgery, The Second Hospital Shanxi Medical University Taiyuan China; ^2^ Department of Nephrology The Second Hospital of Shanxi Medical University Taiyuan China; ^3^ Thyroid surgery department First Hospital of Shanxi Medical University Taiyuan China; ^4^ Shanxi Medical University Taiyuan China

**Keywords:** differentially expressed genes, hub genes, network analysis, neutrophils, venous thromboembolism, weighted gene co‐expression network analysis

## Abstract

The Finkel‐Biskis‐Jinkins Osteosarcoma (c‐Fos; encoded by *FOS*) plays an important role in several cardiovascular diseases, including atherosclerosis and stroke. However, the relationship between FOS and venous thromboembolism (VTE) remains unknown. We identified differentially expressed genes in Gene Expression Omnibus dataset, GSE48000, comprising VTE patients and healthy individuals, and analysed them using CIBERSORT and weighted co‐expression network analysis (WGCNA). FOS and CD46 expressions were significantly downregulated (FOS *p* = 2.26E‐05, CD64 *p* = 8.83E‐05) and strongly linked to neutrophil activity in VTE. We used GSE19151 and performed PCR to confirm that *FOS* and *CD46* had diagnostic potential for VTE; however, only *FOS* showed differential expression by PCR and ELISA in whole blood samples. Moreover, we found that hsa‐miR‐144 which regulates *FOS* expression was significantly upregulated in VTE. Furthermore, *FOS* expression was significantly downregulated in neutrophils of VTE patients (*p* = 0.03). RNA sequencing performed on whole blood samples of VTE patients showed that FOS exerted its effects in VTE via the leptin‐mediated adipokine signalling pathway. Our results suggest that *FOS* and related genes or proteins can outperform traditional clinical markers and may be used as diagnostic biomarkers for VTE.

## 1 INTRODUCTION

Pulmonary embolism, also known as part of venous thromboembolism (VTE), is a type of blood clot forming in the pulmonary arteries.^1^ Every year, between 350,000 and 600,000 people are affected by VTE in the United States, with up to 100,000 related deaths.^2^ VTE can be asymptomatic owing to its small size and distal location. Thus, it is important to identify specific biomarkers for the early detection and diagnosis of VTE. D‐dimer is the primary clinical biomarker for identifying VTE^3^; however, its reliability can be affected by several factors, such as trauma, pregnancy, inflammation, postsurgical status, cancer, disseminated intravascular coagulopathy and renal impairment, which result in the low specificity in predicting VTE.^4^ Therefore, a negative D‐dimer level is often used to exclude VTE.^5^ However, a negative D‐dimer (≤500 ng/mL) level cannot exclude VTE if there is a high pretest probability (Z; Wells score).^6^ In addition to D‐dimer levels, several novel biomarkers such as P‐selectin, tissue factor, vWF, plasminogen activator inhibitor type 1 (PAI‐1), LDL/Lp(α) and growth differentiation factor 15 (GDF‐15) are related to VTE.^7^, ^8^, ^9^, ^10^, ^11^, ^12^, ^13^ Apart from these factors, an increasing number of genetic factors are being studied owing to the complex pathophysiology of VTE. For example, the expression of the human neutrophil antigen 3b (HNA‐3b) epitope on the Slc44a2 protein can reduce the risk of venous thrombosis in humans.^14^ Novel genetic factors regulating plasma FVIII and VWF have been reported by Sabater‐Lleal et al.^12^ Further genetic studies may reveal the fundamental mechanisms underlying VTE pathology.

The critical role of FOS has been investigated in several cardiovascular diseases. For example, Saliques et al. found that c‐Fos transcript level in blood leukocytes was a potential cumulative biomarker in patients with atherosclerosis.^15^ FOS is also a potential biomarker for target therapy in prevention of stroke among hypertensive patients. Mu et al. confirmed that FOS has a strong correlation with the occurrence and prognosis of infantile spasm; moreover, lentivirus‐mediated FOS knock‐down significantly increased the oxidative stress level, neuronal apoptosis and inhibited mitochondrial function (*p* < 0.05).^16^ The AP‐1/c‐FOS cytokine‐signalling axis can modulate recombinant TNF‐α leading to a strong upregulation of IL‐6 mRNA expression and protein secretion, thereby promoting vascular calcification.^17^

In this study, our goal was to identify novel genetic factors involved in VTE pathogenesis to facilitate its early diagnosis. Therefore, FOS was confirmed to play a key role in VTE and have a high diagnostic value. We downloaded microarray datasets from the NCBI Gene Expression Omnibus database (NCBIGEO). By observing the expression of immune cell marker genes and the relationship between gene expression level and VTE patients, we identified marker genes which are strongly related to the incidence of deep vein thrombosis (DVT). The reliability of the marker genes was verified using whole blood samples as well as by bioinformatics analysis. Finally, to investigate the functional mechanism of hub genes, we determined the related immune cells and their upstream miRNAs, and explored the downstream regulatory pathways using the transcriptome data. These findings will aid in the identification of reliable indicators for early VTE diagnosis and delivery of precise host‐targeted treatment.

## 2 MATERIALS AND METHODS

### 2.1 Microarray data information

The NCBI Gene Expression Omnibus database (NCBIGEO; http://www.ncbi.nlm.nih.gov/geo) is a public repository of microarray profiles and next‐generation sequencing data. The datasets from the GEO were reviewed and downloaded. Gene expression profiles of patients with VTE and blood samples from healthy donors were used as the selection criteria and did not require approval by the local ethics committee. The data were used in accordance with TCGA database usage regulations. Two gene expression profiles, GSE48000 and GSE19151, were generated using the criteria described above. GSE48000 comprised 40 patients with VTE and 25 healthy controls, while 70 patients with VTE and 63 healthy controls were included in the GSE19151 dataset. The diagnostic criteria for both datasets were clinical history and imaging data. GSE48000 was utilized to filter differentially expressed genes (DEGs) and perform CIBERSORT and weighted gene co‐expression network analysis (WGCNA). Hub genes were validated using the GSE19151 dataset. Appendix S1 depicts a flow chart describing the methodology used in this study.

### 2.2 Discovery of DEGs and functional enrichment studies

The GEO provided two datasets of matrix files. The R package ‘limma’^18^ was used to identify DEGs in VTE and healthy samples. A false discovery rate <0.05, and |log_2_fold change| ≥1 were the cut‐off parameters for screening DEGs. The R package ‘clusterprofiler’^19^ was used to perform gene ontology (GO) enrichment and Kyoto Encyclopedia of Genes and Genomes (KEGG) pathway analyses. With an adjusted *p* value <0.05, GO keywords or KEGG pathways were deemed statistically significant.

### 2.3 Immune cells and hub gene expression analysis

CIBERSORT was used to evaluate 22 immune cell types and quantify the differences in immune cells of patients with VTE and healthy donors. CIBERSORT is a gene signature‐based method that combines the benefits of gene set enrichment with deconvolution.

### 2.4 Weighted co‐expression network analysis

The R package ‘WGCNA’^19^ was used to create an expression network. When the power was 16 (*R* ^2^ = 0.8), the adjacency matrix was converted into a topological overlap matrix. Based on GO and KEGG analyses, the module with the greatest association with immune cell types was selected to investigate its biological function.

### 2.5 Detection of hub genes

Genes showing the highest association with differently expressed immune cells were identified as the hub genes. First, hub genes in the most important module were filtered using the criteria of gene significance >0.5 and module membership >0.8. To compare hub genes and DEGs, Venn analysis (http://bioinformatics.psb.ugent.be/webtools/Venn/) was used to identify common genes.

### 2.6 Validation of the external dataset

Using the dataset GSE19151, the differentially expressed hub genes of healthy donors and patients with VTE patients were verified. GEO was used to derive gene expression profiles. GSE19151 was composed of 70 patients with VTE and 63 healthy controls and differences in hub gene expression of the healthy donors and patients with VTE patients were subsequently compared. Thereafter, using R packages ‘pROC’^20^ and ‘verification’, we created receiver operating characteristic (ROC) curves, calculated the p‐value of hub genes and calculated clinical indicators. Boxplot is drawn by boxplot. The PCA diagram was drawn by R software package ggord. The two‐gene correlation map is implemented by R software package ggstatsplot, and the multi‐gene correlation map is displayed by R software package pheatmap.

### 2.7 Interaction of genes and miRNAs

ENCORI website (https://rnasysu.com/encori/) was used to identify upstream miRNAs regulating hub genes.^21^ GSE24149 was used to identify the differently expressed miRNAs between VTE and normal patients.

### 2.8 Validation of hub genes

The study protocol was approved by the Ethics Committee of the Second Hospital of Shanxi Medical University and complied with the ethical principles outlined in the Declaration of Helsinki.

#### 2.8.1 Blood sample collection

Peripheral venous blood samples were collected into EDTA tubes from seven patients with acute deep vein thrombosis (DVT) of the lower extremities and seven healthy controls with matched age, sex and comorbidities. Among these, six whole blood samples (from three patients with VTE and three controls) and eight neutrophil samples were used to detect the expression of *FOS* and *CD46*. Another six whole blood samples (from three patients with VTE and three controls) were used to perform RNA isolation and microarray hybridization.

#### 2.8.2 Neutrophil purification

Circulating neutrophils were isolated using Percoll density gradient. Following centrifugation at 500*g* for 30 min at 25 °C, the upper interface containing the neutrophil fractionwas collected. Erythrocytes were removed using red blood cell lysis buffer (TBDsciences, Tianjin, China), the remaining neutrophil fractions were washed three times with washing buffer. The pelleted cells were resuspended in 1 mL phosphate‐buffered saline. The number of neutrophils was determined by Neubauer chamber counting and the purity was assessed by performing Wright‐Giemsa staining. Flow cytometry was performed to further determine the purity of extracted neutrophils using BD FACS Aria II (BD Biosciences, NJ, USA), and the results were analysed using FlowJo 10.8 software (Treestar, USA). Relevant antibodies were purchased from BioLegend (CA, USA).

#### 2.8.3 Quantitative real‐time PCR to determine FOS, CD46, LEPR and hsa‐miRNA‐144 expression in whole blood and neutrophils

The RNA‐Quick Purification Kit and M5 Liquid Sample Total RNA Extraction Reagent (Servicebio, Wuhan, China) were used to extract total RNA from peripheral blood and neutrophils, respectively. Nanodrop 2000 was used to determine the concentration and purity of total RNA (Thermo Scientific Inc., Waltham, MA, USA). Total RNA was reverse‐transcribed to cDNA using the Servicebio®RT First Strand cDNA Synthesis Kit (Servicebio), and quantitative real‐time polymerase chain reaction (qRT‐PCR) was performed using the 2SYBR Green qPCR Master Mix (Servicebio). β‐Actin, GAPDH and U6 were used as internal controls for normalizing mRNA and miRNA expression, respectively. Appendix S2 lists the primers used in this study. The comparative Ct method (2^−∆∆Ct^ method) was used to calculate the relative mRNA expression after normalization. The information was reported as a fold‐change in expression compared to that in normal tissues. For comparison, a one‐way analysis of variance was used, with *p* < 0.05 indicating a statistically significant difference.

#### 2.8.4 Validation of FOS protein in peripheral blood

Venous blood was centrifuged at 200*g* for 20 min at 4°C, and serum was collected and stored at − 80°C. FOS level in blood or serum was determined using a human FOS enzyme‐linked immunosorbent assay (ELISA) kit (Sangon Biotech, China). The minimum detectable dose of FOS was 0.1 ng/mL, and the range of the kit ranged from 0.25–0.8 ng/mL.

#### 2.8.5 RNA isolation and microarray hybridization

Blood was collected in EDTA tubes, and RNA was isolated using the Paxgene Blood RNA and total RNA extractor (TRIzol) kits (Sangon). RNA quality was checked by using a Qubit RNA Detection kit (Life, Santa Clara, CA, USA). All steps involved in RNA processing, probe preparation, microarray hybridization and data processing were performed on the Novaseq6000 platform.

### 2.9 Cell culture and differentiation

The human HL‐60 cell line (iCell, China, iCell‐h098) has emerged as a valuable model system for studying neutrophils. To differentiate into neutrophil‐like cells (dHL‐60), 1 × 106 cells/mL HL‐60 cells were incubated with 1% dimethyl sulfoxide (DMSO, Sigma‐Aldrich, St Louis, MO, USA) for 72 h. The dHL‐60 cells were then determined by Romanowsky Stain. MiR‐144 inhibitor and miR‐144 mimics were used to reduce and enhance the functions of miR‐144, respectively. NC inhibitors or NC mimics were the negative controls. We also use siFOS and novagen to decrease and increase expression of FOS. 200 μL jetPRIME buffer was added into EP tube, and mimics 70 or 35 nM inhibitor was added. For FOS, we added 2 μg plasmid or 15 nM siRNA. 4 μL jetPRIME reagent and buffer was mixed and incubated for 10 min at room temperature. Above jetPRIME reagent and buffer were joined in cell cultures and medium was replaced with fresh one after 6 h. Follow‐up tests were performed 48 h after transfection. The mimics sequence was as follow: Sense 5′‐UACAGUAUAGAUGAUGUACU‐3′; Anti‐sense 5′‐UACAUCAUCUAUACUGUAUU‐3′. The inhibitor sequence was as follow: 5′‐AGUACAUCAUCUAUACUGUA‐3′. Table 1 contains the primer sequences for detection.

**TABLE 1:** siRNA sequences for knockdown FOS.

siRNA	siRNA sequences
FOS‐1	Sense: 5′‐ CGGGCUUCAACGCAGACUACG‐3′
	Anti‐sense: 5′‐ UAGUCUGCGUUGAAGCCCGAG‐3′
FOS‐2	Sense: 5′‐ GGAGACAGACCAACUAGAAGA ‐3′
	Anti‐sense:5′‐ UUCUAGUUGGUCUGUCUCCGC ‐3′
FOS‐3	Sense: 5′‐ GGGAUAGCCUCUCUUACUACC ‐3′
	Anti‐sense: 5′‐ UAGUAAGAGAGGCUAUCCCCG ‐3′
siRNA‐NC	Sense: 5′‐ UUCUCCGAACGUGUCACGUTT‐3′
	Anti‐sense: 5′‐ ACGUGACACGUUCGGAGAATT ‐3′

### 2.10 Advanced analysis of single‐cell data based on Seurat

The Seurat packet (https://satijalab.org/seurat/) in R was then used for cell filtration and analysis. We set the condition of cell filtration as: the number of genes identified in single cells greater than 500 and fewer than 30,000; the total number of UMI detected in a single cell greater than 1000; the proportion of mitochondrial gene expression in single cells less than 25%; use the DoubletFinder package to remove multicellular cells. The follow‐up for further filtering, standardization, cell subgroup classification, each subgroup analysis and marker gene screening of differentially expressed genes was performed by Seurat.

## 3 RESULTS

### 3.1 Identification of stable DEGs and their functions

**FIGURE 1:**
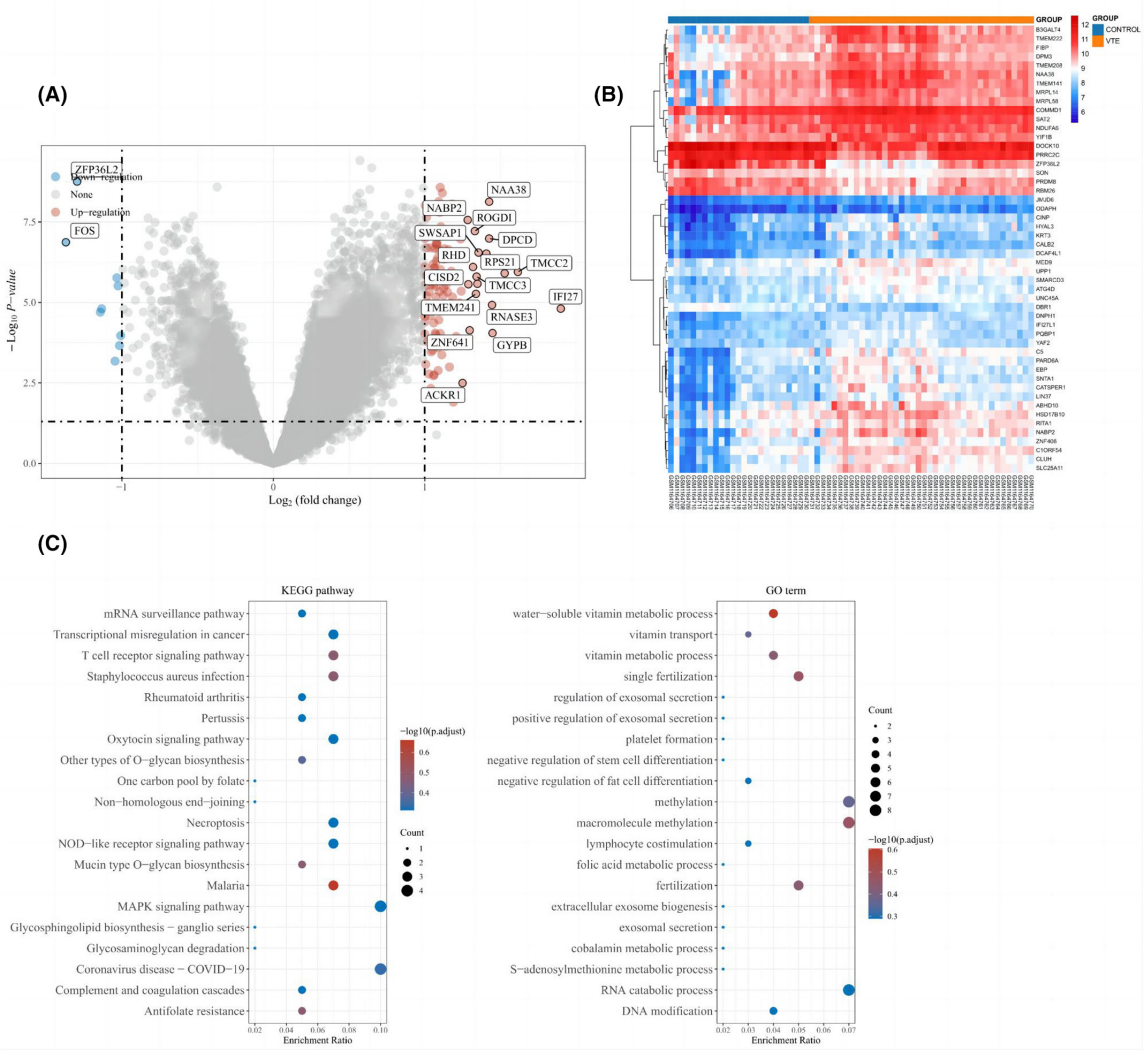
Differentially expressed genes and their enrichment functions. (A) Upregulated DEGs are indicated in red and downregulated DEGs are indicated in blue. (B) The top 50 genes are based on fold change in their expression. (C) GO and KEGG functional enrichment analysis of DEGs.

The DEGs of VTE patients and healthy individuals were determined using the ‘limma’ package in R. Expression of nine genes was substantially downregulated whereas that of 111 genes was significantly upregulated (*p* < 0.05) (Figure 1A). Detailed information on *p* value and fold change about all DEGs were shown in Appendix S3. Among the downregulated genes, *FOS* expression was significantly downregulated (*p* = 2.26E‐05). The heatmap in Figure 1B shows the top 50 DEGs. These genes were mostly identified in KEGG pathways related to signalling via T‐cell and Toll‐like receptors, both of which are implicated in immunological and inflammatory responses (Figure 1C).

### 3.2 Linking of the immune system topography to VTE symptoms

**FIGURE 2:**
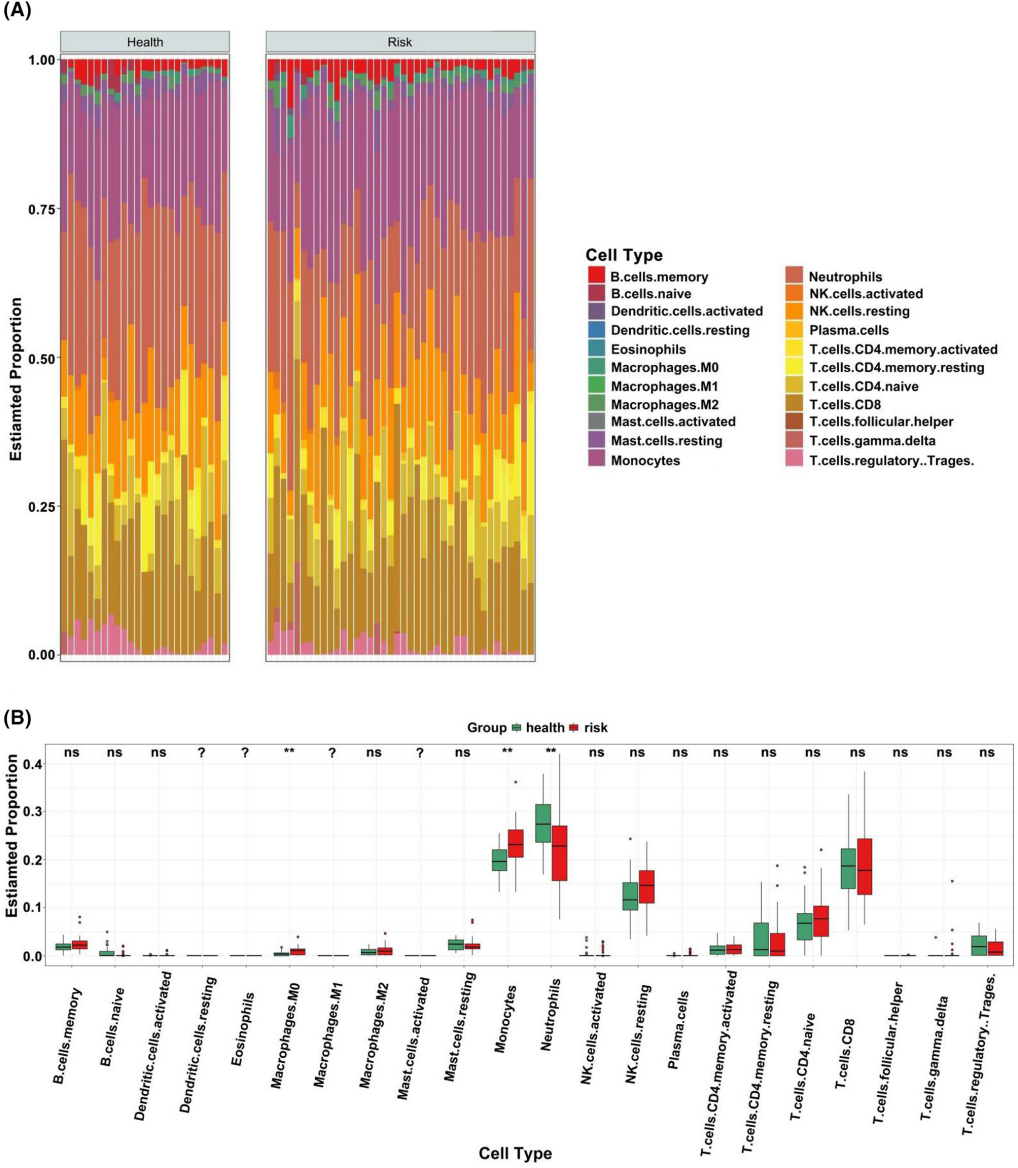
(A) Distribution of immune cells in each sample (B) The differential expression of each type of immune cell among patients with VTE and normal individuals, displayed in red and green, respectively.

To study the *FOS* expression pattern in immune cells and its association with VTE development, we used microarray data derived from 65 whole‐blood samples available in the GSE48000 dataset. Detailed information for different expressions was shown in Appendix S4. Based on the distinct gene markers of normal controls and VTE samples, CIBERSORT analysis revealed specific enrichment for 22 immune cell types. Counts of neutrophils, M0 macrophages and monocytes were significantly different between normal controls and VTE patients (*p* < 0.05). Among them, neutrophil counts were decreased and M0 macrophage and monocyte counts were increased significantly in patients with VTE (*p* < 0.01) (Figure 2B).

### 3.3 Modules identification

**FIGURE 3:**
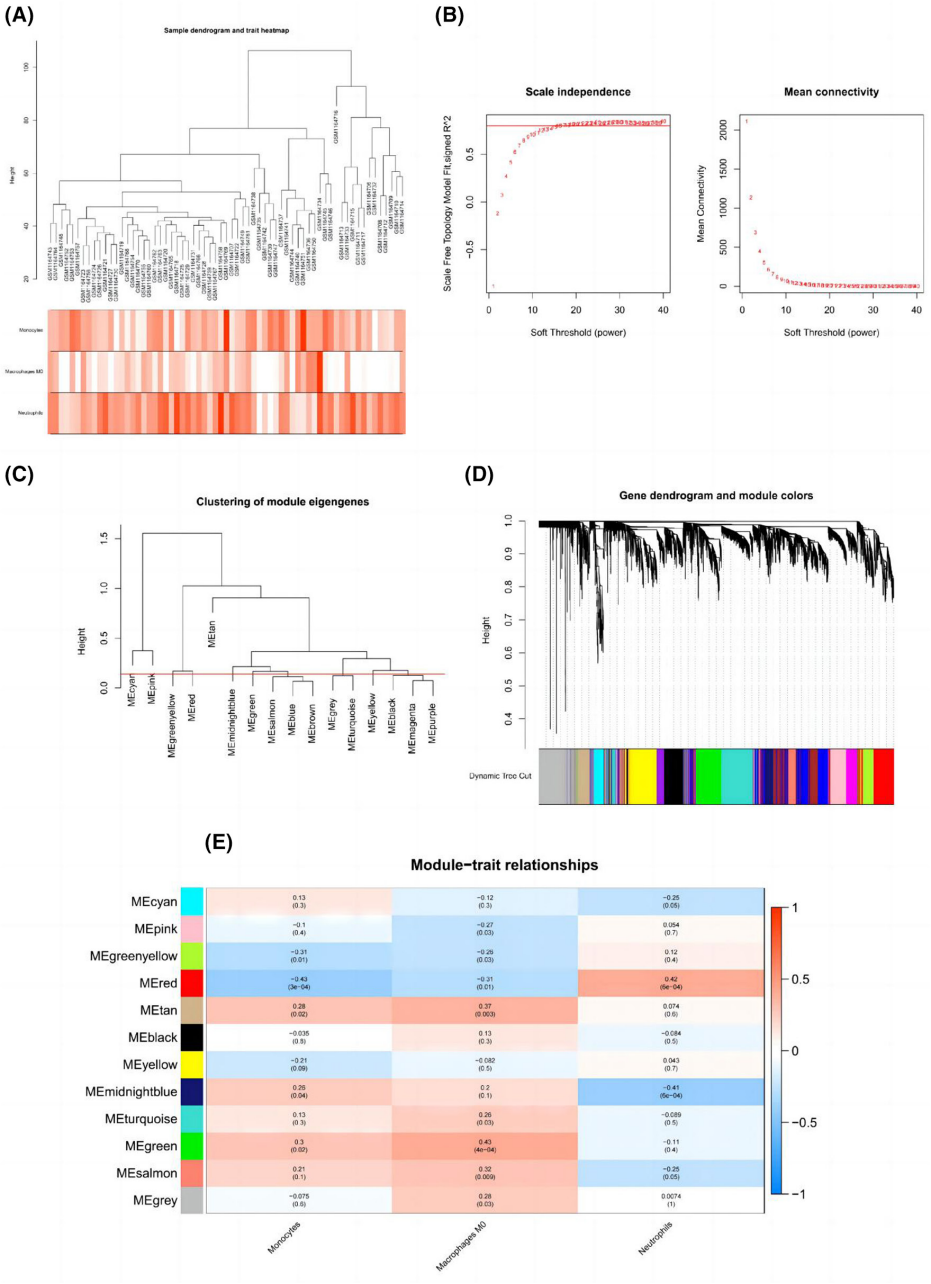
Weighted immune‐cell co‐expression network analysis. (A) Dendrogram of the samples and a heat map of the traits (GSE48000). (B) Analysis of scale‐free fit index (left) and mean connectivity (right). (C) Module eigengene clustering with the cut‐off height indicated by the red line (0.14). (D) Use of a dissimilarity measure (1‐TOM) to cluster gene dendrograms. (E) Module trait connections evaluated using correlations between module eigengenes and sample characteristics.

The GSE48000 dataset was used to build the co‐expression network using WGCNA to identify the main modules associated with VTE. Raw files were used to extract various cell types, such as neutrophils, M0 macrophages and monocytes. We identified 12 modules (Figure 3D) by setting the soft‐thresholding power to 16 (scale‐free *R* ^2^ = 0.8) (Figure 3A,B) and height cutoff to 0.14 (Figure 3C). The correlation between module eigengene values and sample characteristics, which are depicted using heatmap profiles, was used to evaluate the link between modules and clinical aspects of the samples (Figure 3E). Monocytes‐red was the most closely adjusted module (Pearson coefficient = −0.43, *p* = 3E‐04), followed by M0 macrophages‐green (Pearson coefficient = 0.43, *p* = 4E‐04), neutrophils‐red module (Pearson coefficient = 0.42, *p* = 6E‐04) and neutrophils‐midnight blue (Pearson coefficient = −0.41, *p* = 6E‐04) (Figure 3). We used GO and KEGG analyses to determine the genes that may be used to determine the immune‐related mechanism of VTE. Appendix S5 displays the enrichment of GO keywords in the biological processes, cellular components and KEGG pathways. The enrichment results of both functional and pathway analyses were closely related to pyroptosis and the inflammatory effect of NO.

### 3.4 Discovery and validation of FOS playing a critical role in neutrophil

**FIGURE 4:**
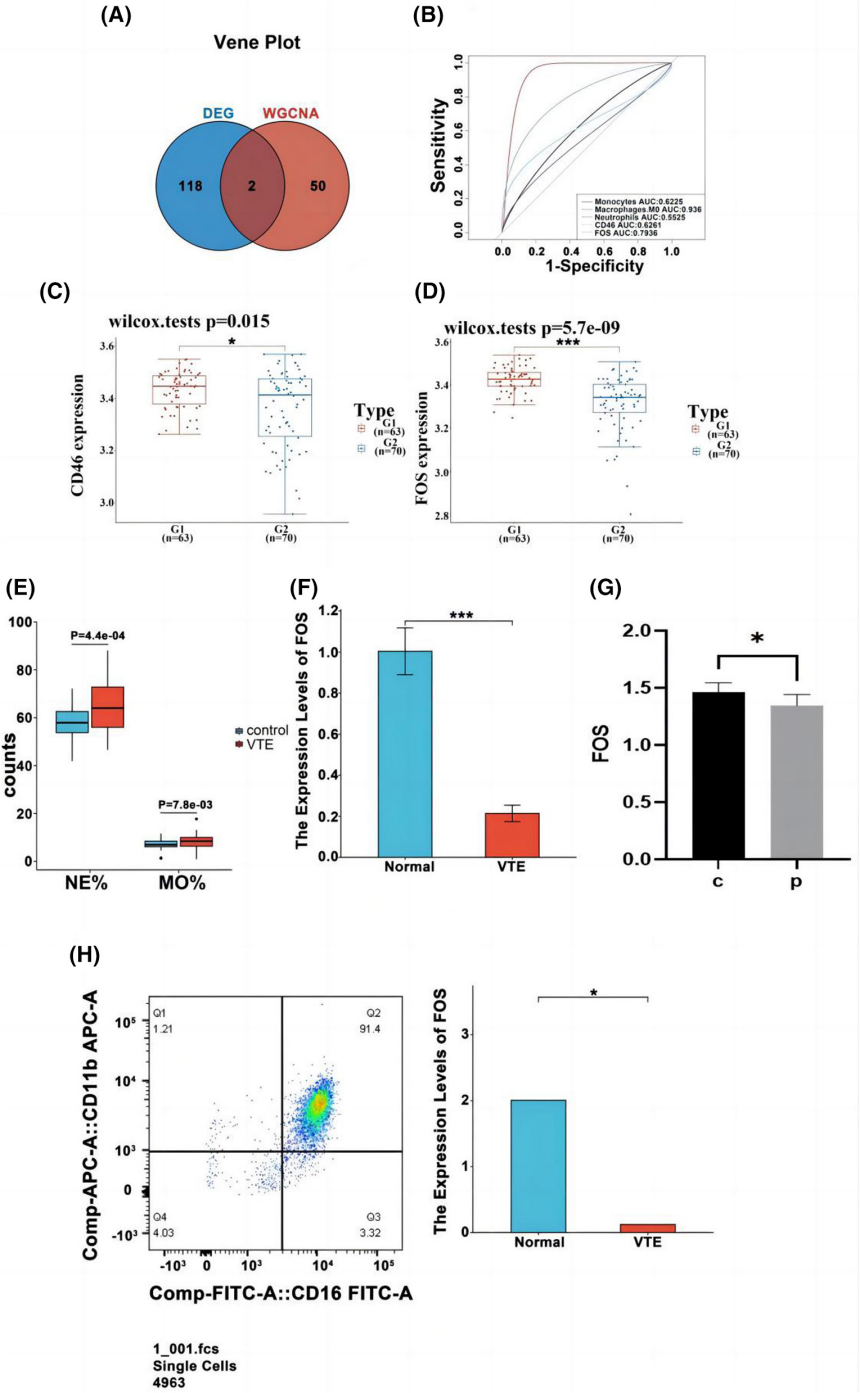
(A) Venn plot indicating overlapping of two commonly modulated genes, and identification of genes at the intersection based on the module. (B) VTE prediction using FOS and CD46 expression levels, proportion of neutrophils, M0 macrophages and monocytes. (C, D) Validation of hub genes in the data set GSE19151 FOS and CD46 showed highly differential expression in VTE. The diagrams from the validation set compare healthy donors (red, *n* = 63) and patients with VTE (blue, *n* = 70). (E) The relative counts of neutrophils and macrophages in whole blood. (F) RT‐PCR and ELISA validation of FOS expression among patients with VTE and normal controls. (G) Flow cytometry results showed 91.4% neutrophils in extracted cells. (H) RT‐PCR validation of FOS expression in neutrophils among patients with VTE and normal controls.*p < 0.05; **p < 0.01; ***p < 0.001.

To screen stable and robust hub genes, 52 genes with significant associations and module membership were selected with module membership cutoff >0.8 and gene significance cutoff >0.5 (Appendix S6). Importantly, *FOS* was one of two commonly altered genes that were shared by hub genes in the neutrophil module and DEGs (Figure 4A).

Both genes (FOS and CD46) showed a consistent positive correlation with neutrophils (Figure 3E). The ROC curves also demonstrated their utility as diagnostic biomarkers for VTE. ROC analysis of the area under the curve (AUC) revealed that the expression levels of *FOS* and *CD46* and the proportion of neutrophils, M0 macrophages and monocytes characterized VTE (Figure 4B). We collected the whole blood from 80 normal individuals and 54 patients and statistically analysed the relative counts of neutrophils and macrophages. The numbers of neutrophils and macrophages were significantly different between VTE patients and healthy individuals (neutrophils *p* = 4.4E‐04; macrophages *p* = 7.8E‐03) (Figure 4E). Another dataset (GSE19151) was analysed using PCR to validate the significantly different expression levels of FOS and CD46 (*p* < 0.05) (Figure 4C,D). Whole‐blood PCR (*p* < 0.0001) and ELISA results (*p* < 0.05) showed that *FOS* expression was significantly downregulated (Figure 4F), whereas CD46 expression was not significant (Appendix S7). Therefore, *FOS* was confirmed to be differentially expressed in the neutrophils of VTE patients. Flow cytometry analysis revealed a 91.4% neutrophil proportion (Figure 4G), while PCR showed that *FOS* expression was significantly downregulated in neutrophils (*p* < 0.05), which was consistent with the expression trend observed in whole blood (Figure 4H). Meanwhile, we analysed the correlation of common thrombosis markers like P‐selectin, tissue factor, vWF, plasminogen activator inhibitor type 1 (PAI‐1), LDL/Lp(α) and growth differentiation factor 15 (GDF‐15) and found that FOS was negatively correlated with all thrombus markers, and significantly correlated with SELP and VWF (Appendix S8). This is also consistent with the significant downregulation of FOS in deep vein thrombosis.

**FIGURE 5:**
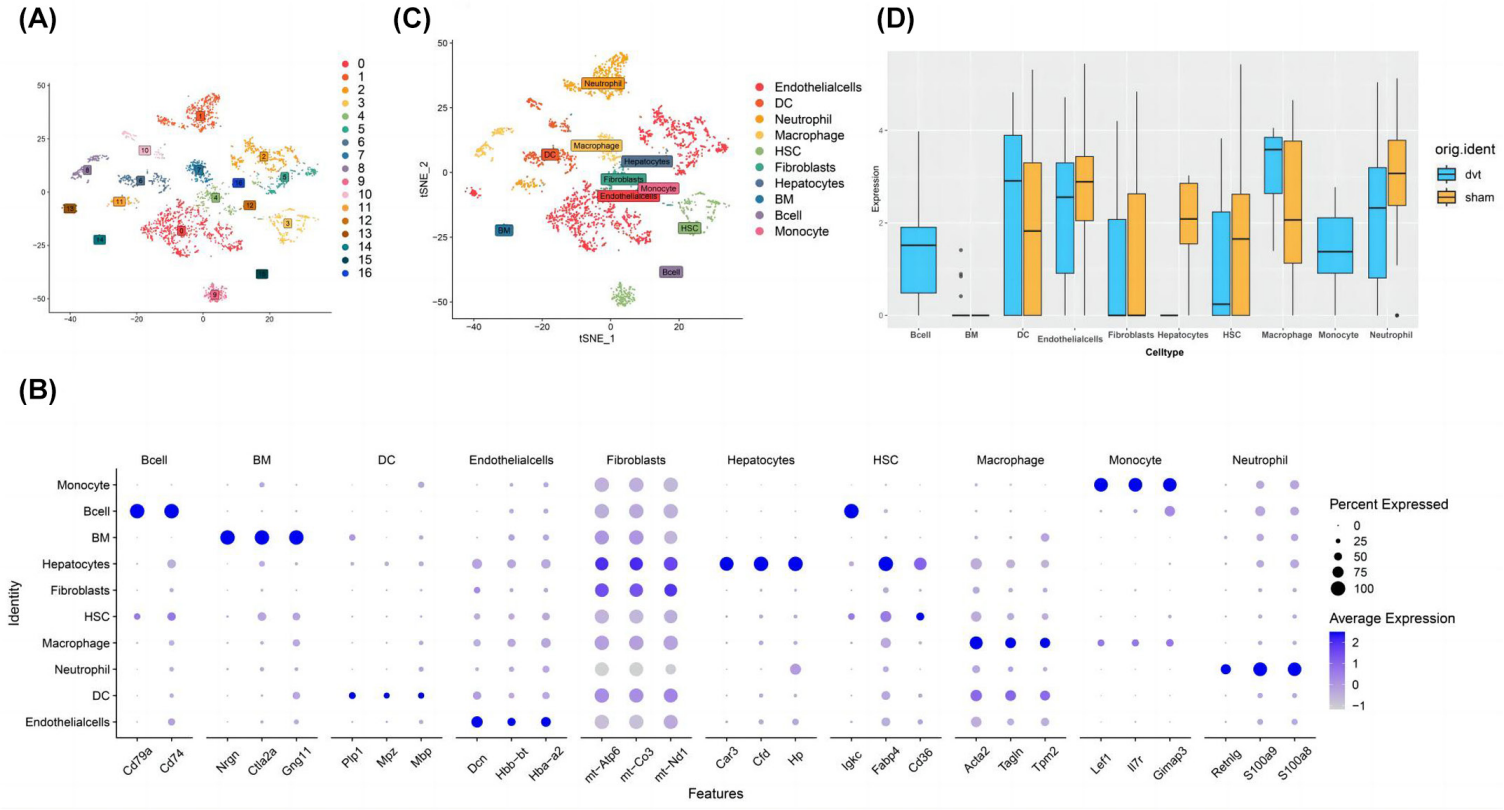
(A) The tSNE projection cluster scatter diagram was noted with clusters. (B) The clusters and the marker genes were noted in the dotplot. (C) The tSNE projection cluster scatter diagram was noted with cell types. (D) The FOS expression of DVT and normal group in each cell type.

To further confirm different expression of FOS in neutrophils, we performed single‐cell analysis of the normal inferior vena cava (IVC) wall and IVC wall from the murine model of IVC ligation obtained from GSE221978. Two thousand eight hundred and ninety cells from one deep venous thrombosis and one venous wall were taken. All cells' gene expression variability was subjected to principal component analysis, and the cells were subsequently divided into cell‐type groups using graph‐based clustering of the informative principal components. Seventeen groups were found using graph‐based clustering, as shown in Figure 5A. The top five expressed genes (fold change) in each cluster were matched with known indicators. Thus, we discovered clusters of cells that could be simply ascribed to established cell lineages using standard marker genes. We identified 10 cell types, including Endothelial cells, DC, Neutrophils, Macrophages, HSCs, Fibroblasts, Hepatocytes, BM, B cells and Monocytes (Figure 5B). The tSNE cluster plot is shown in Figure 5C.

We further analysed the different expression of FOS between DVT and normal venous walls in different cell types and found that in all cell types, neutrophils not only showed high expression of FOS but also expressed less FOS in DVT group, which was the same as the results of the above bulk RNA sequencing.

### 3.5 FOS is a target of hsa‐miR‐144

**FIGURE 6:**
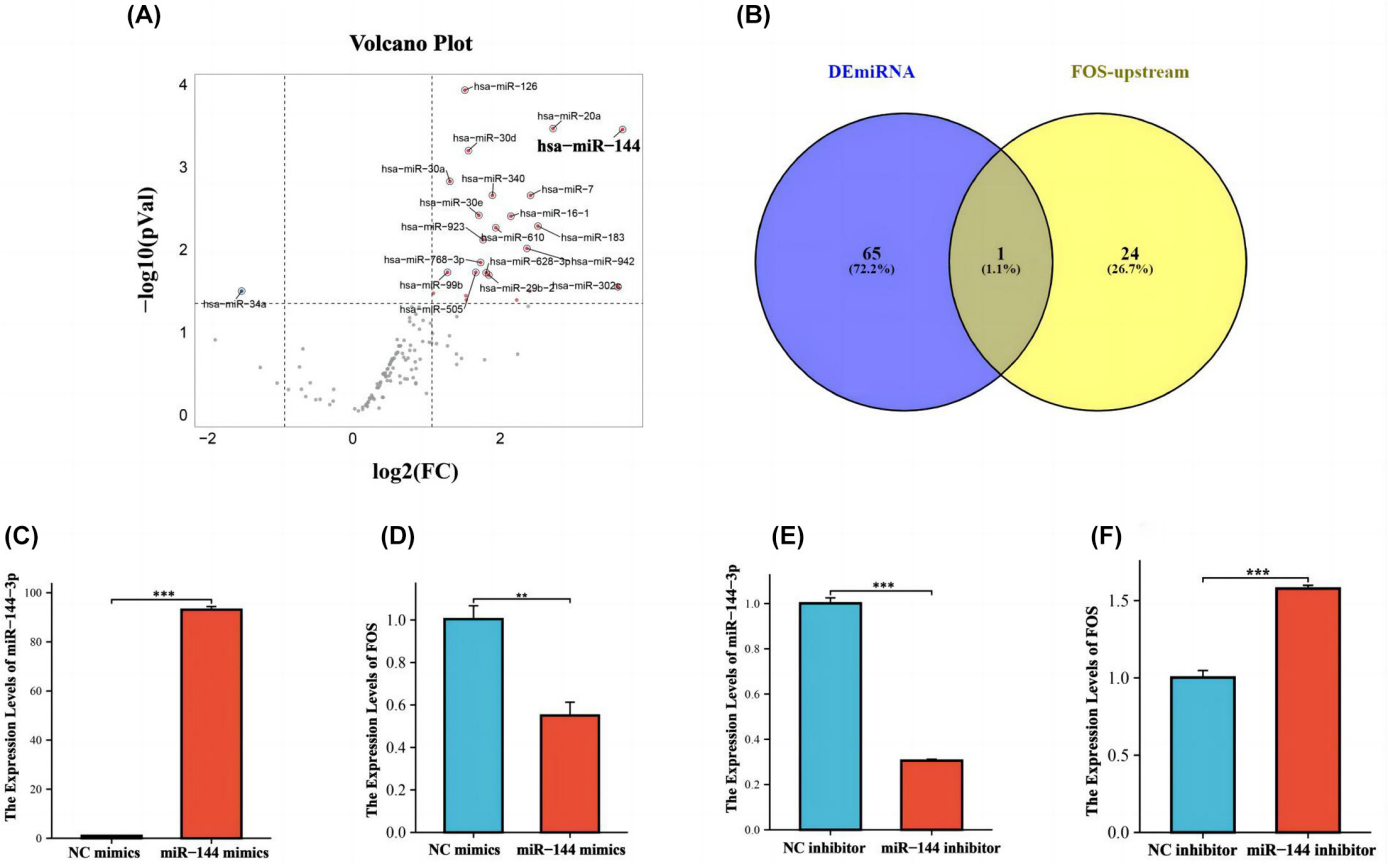
(A) The differentially expressed miRNAs between VTE patients and normal samples are represented in a volcanic map. hsa‐miR‐144 is labelled in the figure. (B) A Venn diagram was used to identify the microRNA that was not only differently expressed between the VTE patients and normal people but also the upregulator of FOS. DEmiRNA: Differently expressed miRNAs. (C) qRT‐PCR was conducted to verify the expression of miR‐144‐3p in dHL‐60 cells transfected with independent shRNA targeting miR‐144‐3p. (D) Expression of FOS was detected by qRT‐PCR in response to the knockdown of miR‐144‐3p. (E) qRT‐PCR was applied to detect the expression of FOS in neutrophils after transfected with miR‐144‐3p vectors for miR‐144‐3p overexpression. (F) Expression of FOS was detected by qRT‐PCR in response to miR‐144‐3p overexpression. *p < 0.05; **p < 0.01.

To identify upstream regulators of FOS, we obtained differentially expressed miRNAs from GSE24149 dataset (Figure 6A) and intersected with FOS upstream miRNAs from the starBASE website. Finally, we found that hsa‐miR‐144 not only differentially expresses but is also the upstream regulator of FOS (Figure 6B).

To determine whether hsa‐miR‐144 targets FOS, we transfected mimics to overexpress miRNA in dHL‐60 cells, the overexpression of hsa‐miR‐144 in neutrophils was verified by qPCR (Figure 6C). The results of qPCR experiment showed that  FOS expression decreased after increasing overexpressing hsa‐miR‐144 in neutrophils (Figure 6D). Similarly, the expression level of FOS increased after the expression of hsa‐miR‐144 was inhibited (Figure 6E,F). Therefore, it suggests that hsa‐miR‐144 can trigger FOS and reduce the level of FOS expression in neutrophils.

### 3.6 Identification of pathways regulating FOS in VTE

**FIGURE 7:**
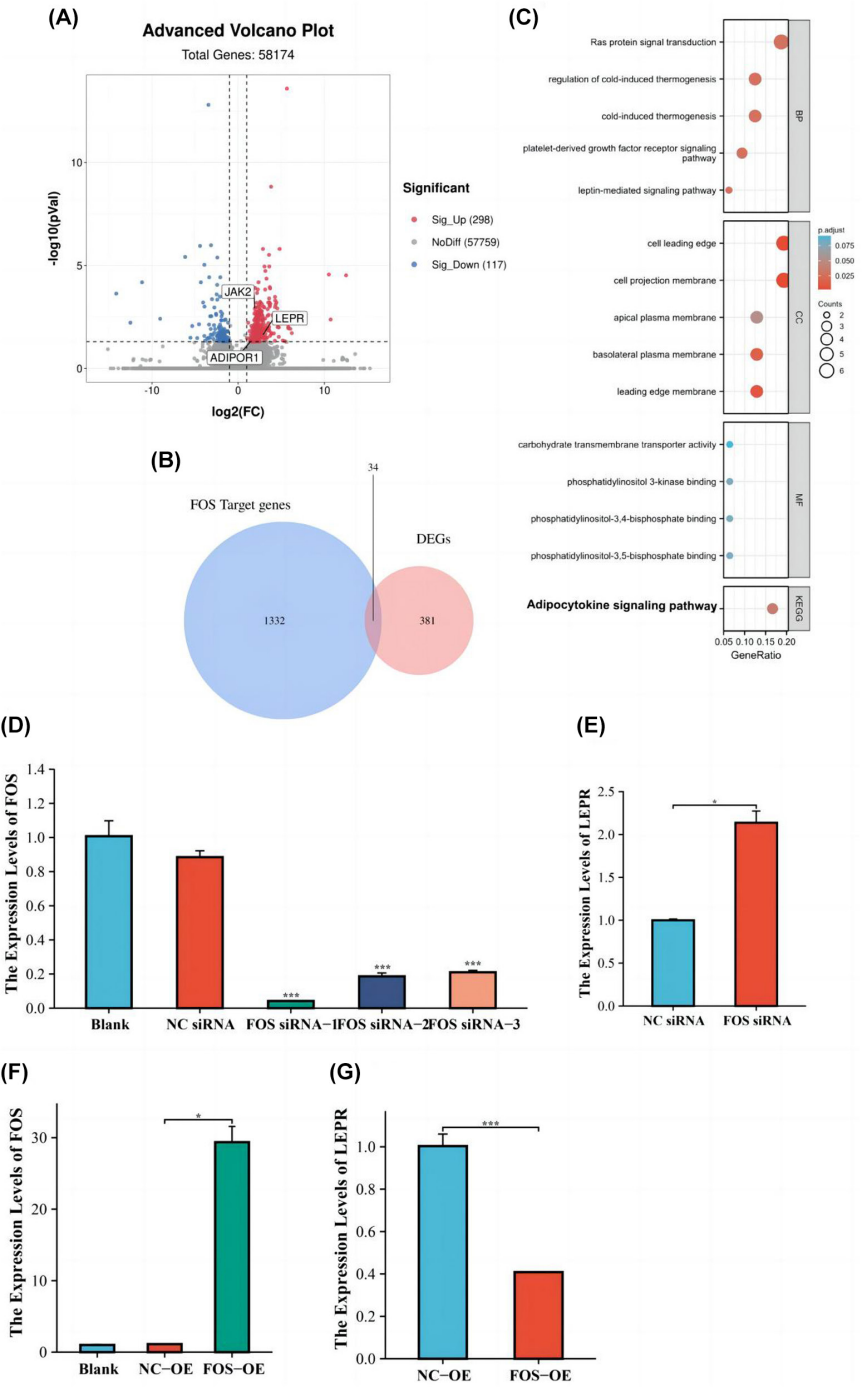
(A) Differentially expressed genes in a private transcriptome sequencing data set: upregulated DEGs are in red and downregulated DEGs are in blue (B) Venn plot indicating overlap of DEGs and FOS‐targeted genes (C) Intersection genes shown as a heat map and sorted by fold change: upregulated genes are indicated in red and downregulated genes are shown in blue. (D) qRT‐PCR was conducted to verify the expression of FOS in neutrophils transfected with three independent shRNA targeting FOS. (E) Expression of LEPR was detected by qRT‐PCR in response to FOS‐knockdown. (F) qRT‐PCR was applied to detect the expression of FOS in neutrophils after transfected with FOS vectors for FOS overexpression. (G) Expression of LEPR was detected by qRT‐PCR in response to FOS‐OE. *p < 0.05; **p < 0.01; ***p < 0.001.

To explore the regulatory pathway of FOS in patients with VTE, we obtained whole blood samples from three VTE patients and three normal individuals, performed sequencing, and used differential expression analysis to determine DEGs. Thereafter, the intersection of DEGs and FOS‐regulated target genes (Appendix S9) was obtained (Figure 7A). Our results suggest that 34 genes may be crucial for mediating FOS effects in VTE (Figure 7B). The 34 intersecting genes were used to perform functional analysis. The results of GO and KEGG analyses showed that the potential mechanism of their action may be related to the adipocytokine and leptin‐mediated signalling pathways (Figure 7C). Among all DEGs, LEPR was not only significantly increased in VTE but also plays a critical role in adipocytokine and leptin‐mediated signalling pathways (Figure 7D). To further investigate the mechanism of FOS regulating the pathway, we next verified whether FOS can regulate the expression of LEPR in neutrophils. For this aim, we introduced three siRNA molecules targeting FOS into neutrophils induced from dHL‐60 by DMSO, and the knockdown efficiency was validated by qRT‐PCR (Figure 7A). Knockdown of FOS increases expression of LEPR (Figure 7E). Meanwhile, we overexpressed FOS in neutrophils (Figure 7F) and found that LEPR expression is significantly decreased (Figure 7G). Collectively, FOS can negatively regulate the expression of LEPR in neutrophils.

## 4 DISCUSSION

Owing to the small size of the thrombus and mild early clinical symptoms, VTE can be easily missed, and thus, move to pulmonary capillaries with the blood flow, forming a pulmonary embolism and leading to death.^3^ To date, no specific and reliable biomarkers have been determined for early detection of VTE. Meanwhile, FOS, an integral part of leukocyte transcriptome, is closely associated with a heightened risk of potentially fatal thrombosis or formation of blood clots. Therefore, we investigated whether *FOS* is a hub immune‐related gene involved in deep vein thrombosis and explored its potential mechanisms of action.

To our knowledge, this is the first study to perform DEG analysis in conjunction with WGCNA method and CIBERSORT program to identify related immune cells and novel hub genes associated with immune infiltration in VTE. We found that *FOS* is differentially expressed in VTE patients compared to healthy individuals and is closely related to the infiltration of neutrophils. Next, we used a new GEO dataset and conducted PCR using whole‐blood samples to verify *FOS* expression. After confirming *FOS* expression in peripheral blood, neutrophils were extracted from peripheral blood to check *FOS* expression using PCR. Furthermore, PCR was performed to verify the expression of miRNAs acting upstream of *FOS*, and sequencing was performed to identify the possible mechanism underlying FOS action. We discovered that *FOS* may be an immune‐infiltration related hub gene and is involved in VTE development under miR‐144‐mediated regulation. These findings add to the existing knowledge regarding the molecular mechanisms underlying *FOS* action.

Neutrophils are among the first immune cells to infiltrate thrombi.^22^ Moreover, web‐like chromatin structures, known as neutrophil extracellular traps, play a key role in venous thrombosis.^23^ The neutrophil count in whole blood can be increased by regulating the expression of FOS protein 1 (AP‐1). In cardiovascular diseases, FOS is activated after thrombin acts on endothelial cells and regulates the expression of VEGF, which plays a protective role in endothelial cells.^24^, ^25^ FOS, a transcription factor, primarily activates programs involved in cellular architectural rearrangements and changes in cell function, thereby playing an important role in several diseases.^26^, ^27^ For example, FOS regulates the development of cells destined to form and maintain the skeleton.^28^ FOS is also a regulatory enhancing the proliferation of lymphocytes in rheumatoid arthritis patients.^29^ Therefore, the regulatory role of FOS in DVT requires further investigation. FOS is regulated by upstream miRNAs to promote disease development. For example, Shikama et al. demonstrated that mir‐34a downregulates FOS expression and reduces TNF‐α‐mediated inhibition, leading to suppressed haematopoiesis in myelodysplastic syndromes. FOS expression is regulated by mir‐335‐5p in malignant thyroid carcinoma, indicating its role in tumour development.^22^, ^30^ Our study verified differential miR‐144 expression and the relationship between FOS and miR‐144, thereby providing evidence that miR‐144 possibly plays a role in regulating *FOS* expression in VTE.

In our study, *FOS* expression is associated with the adipocytokine signalling pathway. Leptin, an adipocyte‐derived hormone, plays an important regulatory role as an immunomodulatory factor in maintaining the homeostasis of immune functions via regulating monocytes and neutrophils.^31^ Moreover, experimental studies in mice have demonstrated that leptin promotes both arterial and venous thrombosis.^32^ Therefore, FOS may be associated with leptin‐mediated regulation of the adipocytokine signalling pathway in VTE.

This study had several limitations. DEGs were linked to neutrophils in a substantial proportion of cases. However, all selected patients were on anticoagulant drugs, and consequently, their influence on gene expression could not be ruled out. Thus, more studies including in vivo studies and randomized controlled trials are required to clarify these effects.

In conclusion, our study findings revealed that FOS is strongly correlated to VTE. *FOS* and its encoded protein had high sensitivity and specificity and may reflect disease severity apart from being a diagnostic biomarker. Moreover, *FOS* was identified as an immune‐infiltration‐related hub gene in VTE neutrophils under the regulation of miR‐144 and was associated with the leptin‐adipocytokine signalling pathway. Overall, these findings might help us identify a specific biomarker for VTE and better understand the mechanisms leading to VTE development. This may lead to new discoveries in molecular mechanisms at work in VTE, thereby facilitating its early diagnosis and precise therapy in the future.

## AUTHOR CONTRIBUTIONS

**Lin Zheng:** Conceptualization (equal); formal analysis (equal); validation (equal). **Honglin Dong:** Conceptualization (equal); resources (equal). **Ping Wang:** Conceptualization (equal); resources (equal). **Xiaotong Qi:** Investigation (equal); validation (equal). **Heng Wang:** Investigation (equal); visualization (equal). **Ruijing Zhang:** Formal analysis (equal); resources (equal). **Liying Song:** Data curation (equal); software (equal). **Ruihan Chen:** Data curation (equal); software (equal). **Sheng Yan:** Funding acquisition (equal); supervision (equal). **Wenkai Chang:** Data curation (equal); resources (equal). **Jie Hu:** Funding acquisition (equal); resources (equal). **Yuwen Wang:** Data curation (equal); resources (equal). **Haijiang Jin:** Funding acquisition (equal); resources (equal). **Yongbin Shi:** Data curation (equal); resources (equal). **Zhihui Wu:** Data curation (equal); resources (equal). **Wenbo Zhao:** Data curation (equal); resources (equal). **Peilu Shi:** Data curation (equal); resources (equal). **Qinqin Tian:** Data curation (equal); resources (equal). **Miao Xing:** Data curation (equal); resources (equal).

## FUNDING INFORMATION

This study was supported by the Youth Science and Technology Research Fund (201901D211493).

## CONFLICT OF INTEREST STATEMENT

There are no conflicts of interest to report.

## Supporting information


Appendix S1.

Appendix S2.

Appendix S3.

Appendix S4.

Appendix S5.

Appendix S6.

Appendix S7.

Appendix S8.

Appendix S9.

Data S1.

## Data Availability

The data that supports the findings of this study are available in the supplementary material of this article.
