# Supplementary figures and images for: Contribution of FOS in neutrophils to venous thromboembolism via miR‐144 based on bioinformatic prediction and validation
Source: J Cell Mol Med. 2024 May 31;28(11):e18370. doi: 10.1111/jcmm.18370 (PMC11140234; doi:10.1111/jcmm.18370)

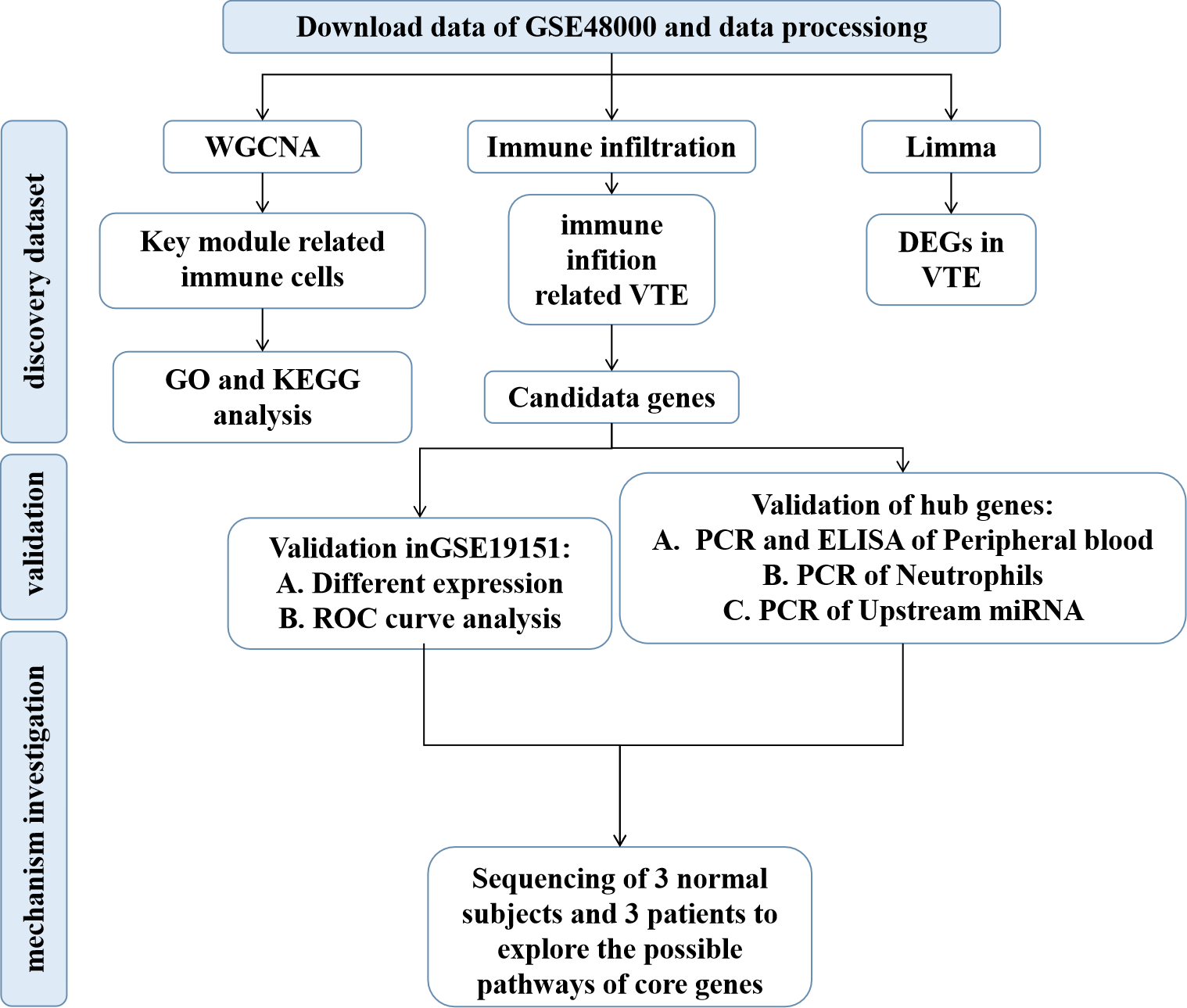

Supplement: Supplementary file 1 — Appendix S1. [file JCMM-28-e18370-s009.tif]

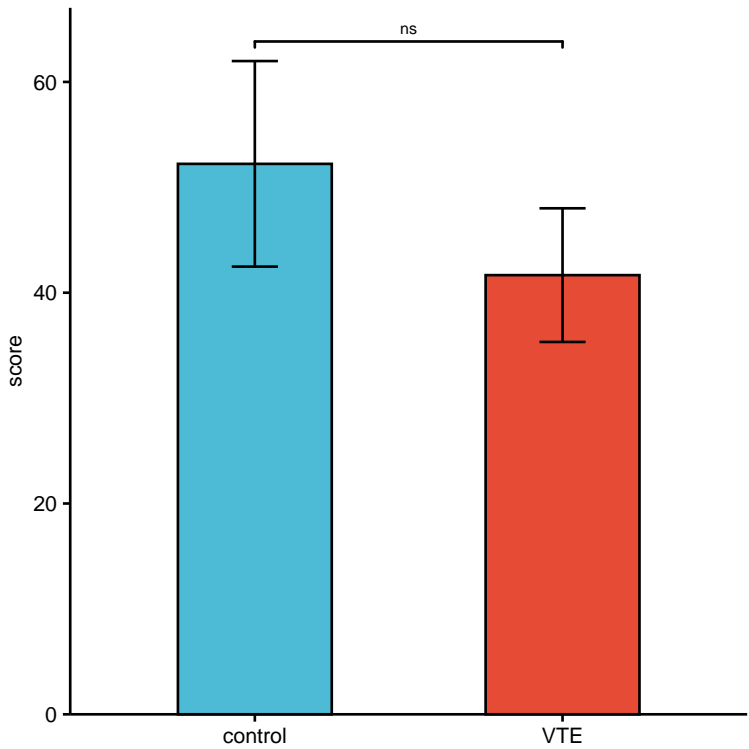

Supplement: Supplementary file 7 — Appendix S7. [file JCMM-28-e18370-s003.zip › supplementary file 7_1.pdf]

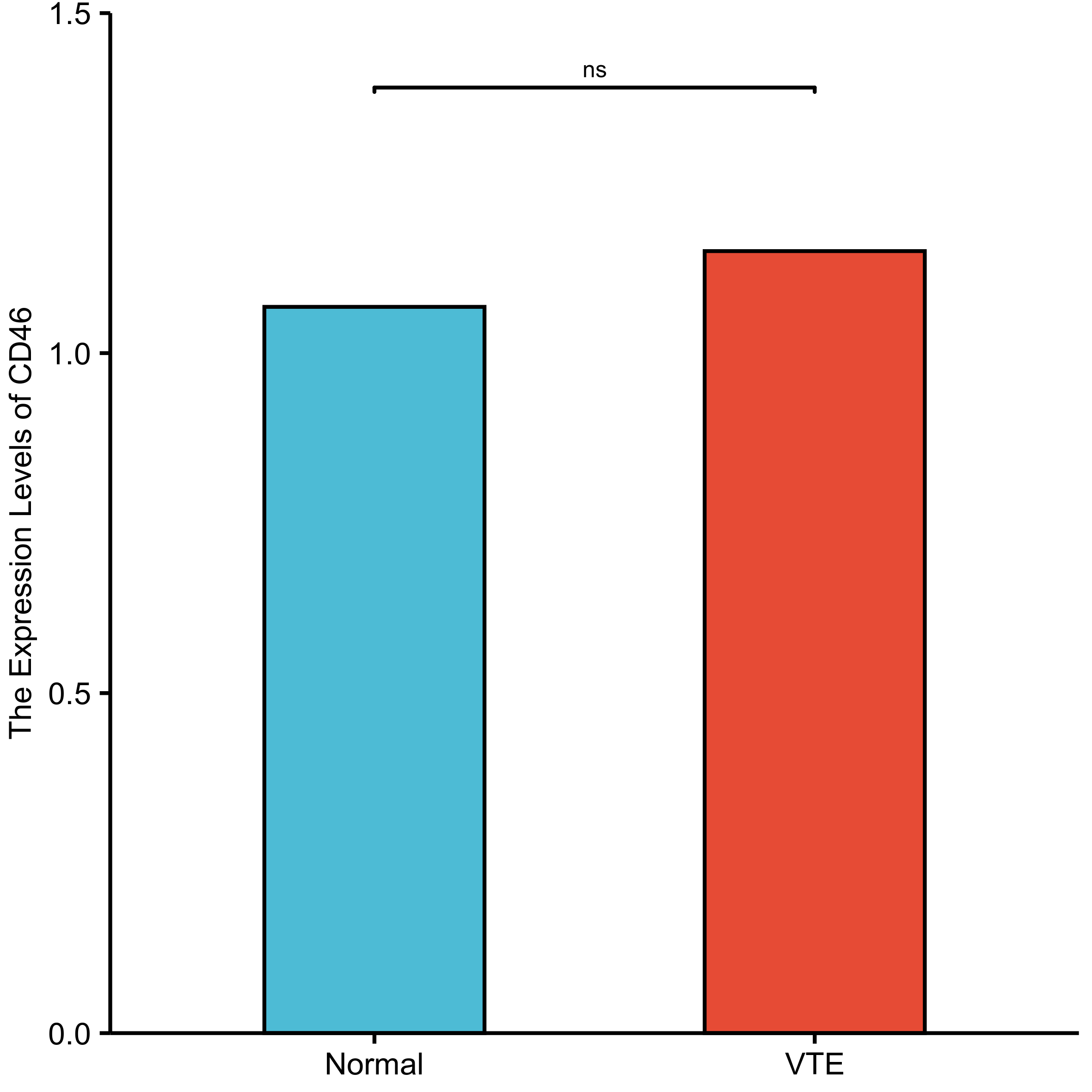

Supplement: Supplementary file 7 — Appendix S7. [file JCMM-28-e18370-s003.zip › supplementary file 7_2.tiff]

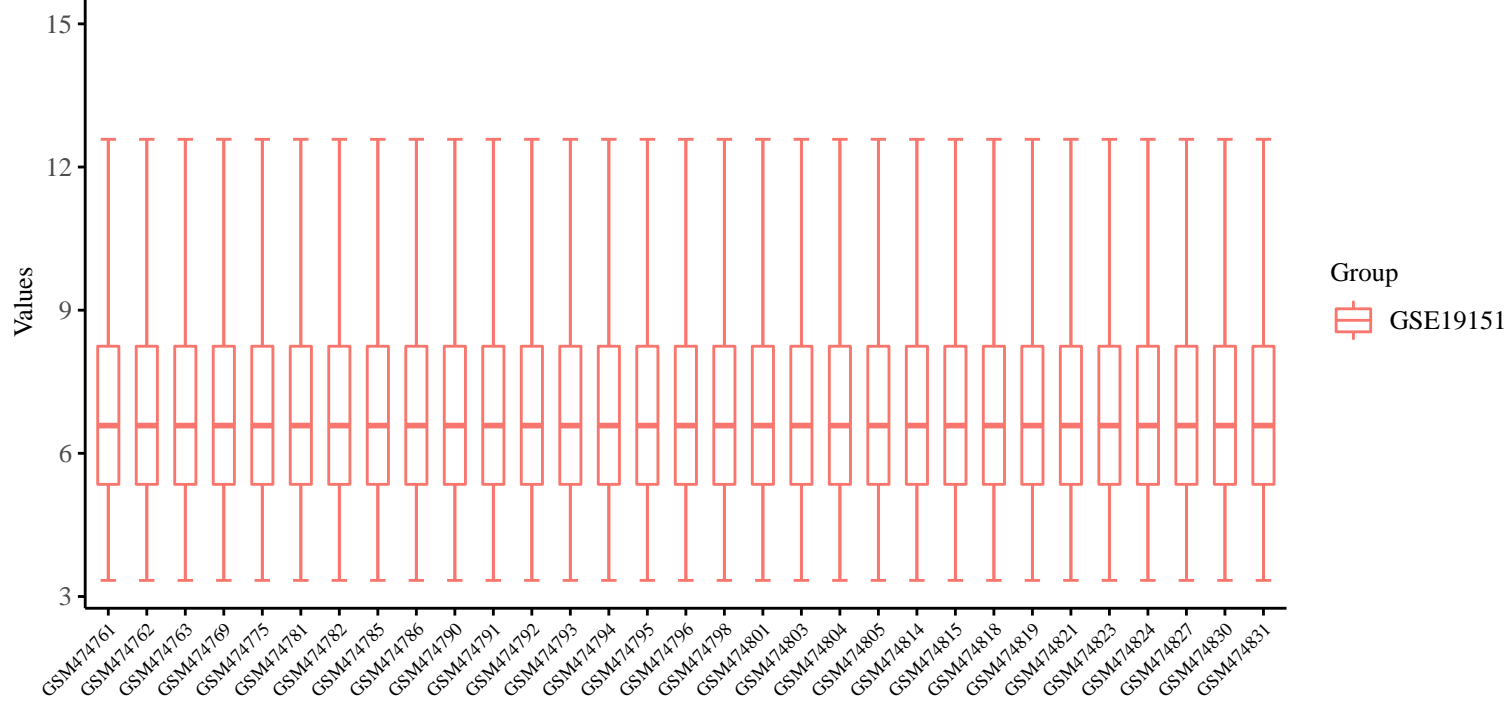

**D**

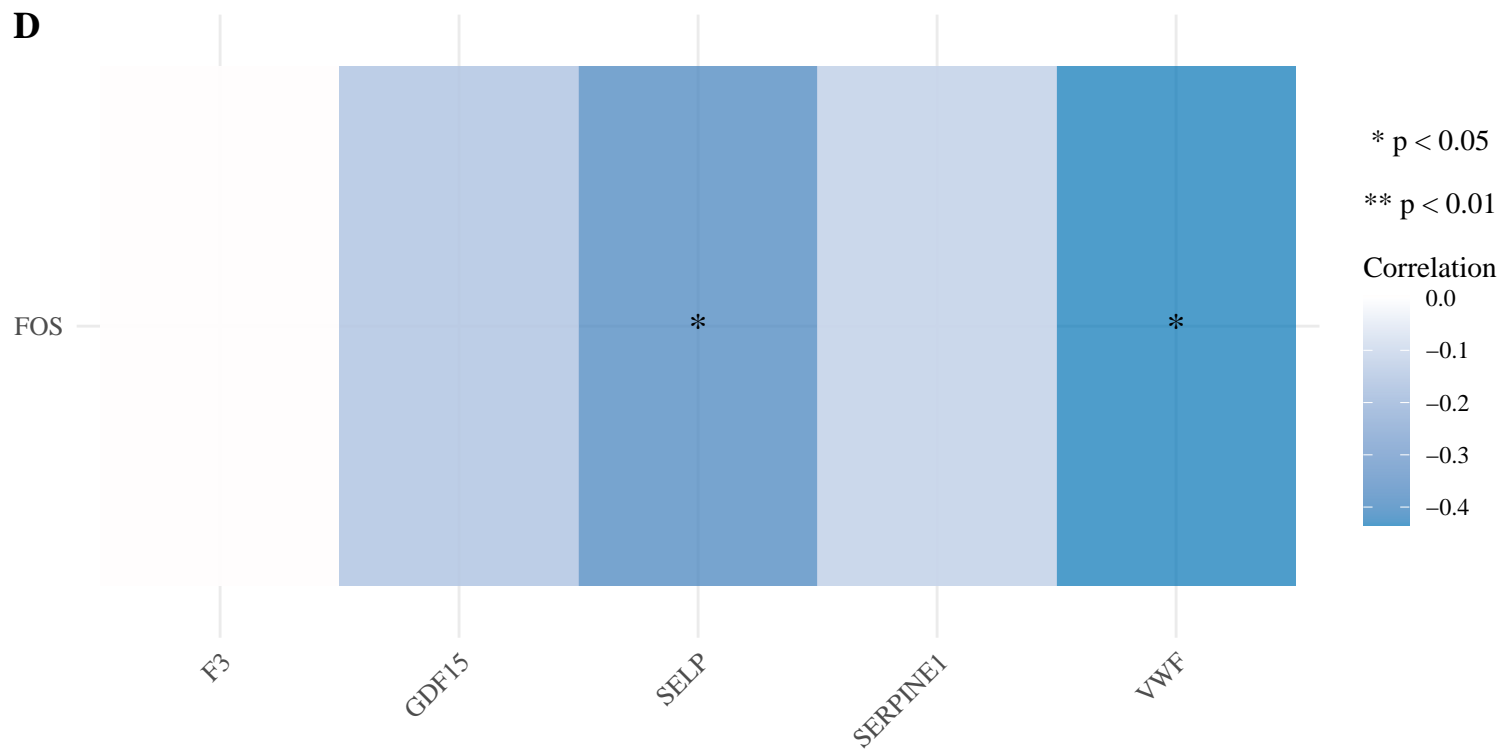

Supplement: Supplementary file 8 — Appendix S8. [file JCMM-28-e18370-s005.pdf]
