# Supplementary material for: Contribution of FOS in neutrophils to venous thromboembolism via miR‐144 based on bioinformatic prediction and validation
Source: J Cell Mol Med. 2024 May 31;28(11):e18370. doi: 10.1111/jcmm.18370 (PMC11140234; doi:10.1111/jcmm.18370)
Supplement: Supplementary file 2 — Appendix S2. [file JCMM-28-e18370-s001.docx]

| Primer name | Primer sequence, 5’-3’ |
| --- | --- |
| FOS-F | TTACTACCACTCACCCGCAGACTC |
| FOS-R | GGGAATGAAGTTGGCACTGGAGAC |
| CD46-F | CGTCCAGTGCCTCAGGTCCTAG |
| CD46-R | ACAGCAATGACCCAAACATCCAAAC |
| Homo GAPDH Forward | TCAAGAAGGTGGTGAAGCAGG |
| Homo GAPDH Reverse | TCAAAGGTGGAGGAGTGGGT |
| Homo LEPR-F | TGCCTCCATCCAGTGTGAAA |
| Homo LEPR-R | CGCACCTGAACAGCATAGAC |
| U6-F | CGCTTCGGCAGCACATATAC |
| U6-R | AAATATGGAACGCTTCACGA |
| hsa-miR-144-primer | GTCGTATCCAGTGCAGGGTCCGAGGTATTCGCACTGGATACGACAGTACATC |
| hsa-miR-144-F | TGCGCTACAGTATAGATGAT |
| hsa-miR-144-R | CCAGTGCAGGGTCCGAGGTATT |
