# Supplementary material for: Contribution of FOS in neutrophils to venous thromboembolism via miR‐144 based on bioinformatic prediction and validation
Source: J Cell Mol Med. 2024 May 31;28(11):e18370. doi: 10.1111/jcmm.18370 (PMC11140234; doi:10.1111/jcmm.18370)
Supplement: Supplementary file 5 — Appendix S5. [file JCMM-28-e18370-s008.docx]

Table A1 Enrichment analysis of genes in immune-cell associated modules

| ONTOLOGY | ID | Description | pvalue | geneID |
| --- | --- | --- | --- | --- |
| BP | GO:0001774 | microglial cell activation | 9.85201E-05 | ATM/NAMPT/LRRK2 |
| BP | GO:0002269 | leukocyte activation involved in inflammatory response | 9.85201E-05 | ATM/NAMPT/LRRK2 |
| BP | GO:0006997 | nucleus organization | 9.94056E-05 | NUP153/CHMP1B/TMEM43/CNEP1R1 |
| BP | GO:0061900 | glial cell activation | 0.000173518 | ATM/NAMPT/LRRK2 |
| BP | GO:0051865 | protein autoubiquitination | 0.000290506 | RNF13/RNF146/LRRK2 |
| BP | GO:0007568 | aging | 0.000309545 | ATM/FOS/NAMPT/ZMIZ1/LRRK2 |
| BP | GO:0051770 | positive regulation of nitric-oxide synthase biosynthetic process | 0.000353035 | NAMPT/LRRK2 |
| BP | GO:0150076 | neuroinflammatory response | 0.000371532 | ATM/NAMPT/LRRK2 |
| BP | GO:0008340 | determination of adult lifespan | 0.000456189 | ATM/LRRK2 |
| BP | GO:0099003 | vesicle-mediated transport in synapse | 0.000587111 | RAB27A/STX3/NUMB/LRRK2 |
| BP | GO:0051767 | nitric-oxide synthase biosynthetic process | 0.000635076 | NAMPT/LRRK2 |
| BP | GO:0051769 | regulation of nitric-oxide synthase biosynthetic process | 0.000635076 | NAMPT/LRRK2 |
| BP | GO:1903978 | regulation of microglial cell activation | 0.000635076 | ATM/LRRK2 |
| BP | GO:0042116 | macrophage activation | 0.000742303 | ATM/NAMPT/LRRK2 |
| BP | GO:0070482 | response to oxygen levels | 0.000786401 | ATM/ATP6V1A/CDKN1B/NAMPT/CYB5R4 |
| BP | GO:0016197 | endosomal transport | 0.000788876 | ACAP2/CMTM6/CHMP1B/LRRK2 |
| BP | GO:1905269 | positive regulation of chromatin organization | 0.000938761 | ATM/RESF1/LRRK2 |
| BP | GO:0002286 | T cell activation involved in immune response | 0.001020131 | CD46/RAB27A/IL18R1 |
| BP | GO:0002456 | T cell mediated immunity | 0.001020131 | CD46/RAB27A/IL18R1 |
| BP | GO:0071156 | regulation of cell cycle arrest | 0.001076785 | ATM/CDKN1B/SDE2 |
| BP | GO:0021591 | ventricular system development | 0.001078672 | NUMB/RAPGEF2 |
| BP | GO:0010256 | endomembrane system organization | 0.001261806 | RAB27A/CHMP1B/TMEM43/LRRK2/CNEP1R1 |
| BP | GO:0007569 | cell aging | 0.001323208 | ATM/NAMPT/ZMIZ1 |
| BP | GO:0036257 | multivesicular body organization | 0.001534284 | RAB27A/CHMP1B |
| BP | GO:0042110 | T cell activation | 0.001627836 | CD46/RAB27A/IL18R1/PELI1/ZMIZ1 |
| BP | GO:0071985 | multivesicular body sorting pathway | 0.001738064 | RAB27A/CHMP1B |
| CC | GO:0005770 | late endosome | 0.000125482 | RAB27A/RAPGEF2/RNF13/CHMP1B/LRRK2 |
| CC | GO:0005771 | multivesicular body | 0.000129025 | RAB27A/CHMP1B/LRRK2 |
| CC | GO:0042581 | specific granule | 0.000248635 | RAB27A/STX3/CMTM6/SLC15A4 |
| CC | GO:0030667 | secretory granule membrane | 0.000254336 | CD46/RAB27A/STX3/CMTM6/SLC15A4 |
| CC | GO:0045177 | apical part of cell | 0.000807463 | ATP6V1A/RAB27A/STX3/NUMB/RAPGEF2 |
| CC | GO:0010008 | endosome membrane | 0.002146888 | RAB27A/RNF13/ACAP2/CMTM6/CHMP1B |
| CC | GO:0031902 | late endosome membrane | 0.002175967 | RAB27A/RNF13/CHMP1B |
| CC | GO:0031965 | nuclear membrane | 0.0024581 | NUP153/RNF13/TMEM43/CNEP1R1 |
| CC | GO:0016324 | apical plasma membrane | 0.003181559 | ATP6V1A/RAB27A/STX3/RAPGEF2 |
| CC | GO:0005766 | primary lysosome | 0.003285501 | STX3/GCA/CMTM6 |
| CC | GO:0042582 | azurophil granule | 0.003285501 | STX3/GCA/CMTM6 |
| CC | GO:0005765 | lysosomal membrane | 0.004659486 | ATP6V1A/RNF13/CMTM6/SLC15A4 |
| CC | GO:0098852 | lytic vacuole membrane | 0.004706129 | ATP6V1A/RNF13/CMTM6/SLC15A4 |
| CC | GO:0005637 | nuclear inner membrane | 0.005585578 | RNF13/TMEM43 |
| CC | GO:0070382 | exocytic vesicle | 0.007342375 | RAB27A/STX3/LRRK2 |
| CC | GO:0005774 | vacuolar membrane | 0.007914918 | ATP6V1A/RNF13/CMTM6/SLC15A4 |
| KEGG | hsa04068 | FoxO signaling pathway | 0.000319468 | ATM/CDKN1B/S1PR1/SGK1 |
