# Supplementary material for: Contribution of FOS in neutrophils to venous thromboembolism via miR‐144 based on bioinformatic prediction and validation
Source: J Cell Mol Med. 2024 May 31;28(11):e18370. doi: 10.1111/jcmm.18370 (PMC11140234; doi:10.1111/jcmm.18370)
Supplement: Supplementary file 6 — Appendix S6. [file JCMM-28-e18370-s002.docx]

Table A2 52 important genes with significant associations

| probes | moduleColor |
| --- | --- |
| green-M0 |  |
| PLBD2 | green |
| GTPBP1 | green |
| FBXL7 | green |
| red-neu |  |
| ZMIZ1 | red |
| TMEM71 | red |
| TMEM43 | red |
| TM2D3 | red |
| SUSD6 | red |
| STX3 | red |
| SLC15A4 | red |
| SGK1 | red |
| SDHD | red |
| SDE2 | red |
| RNF146 | red |
| RNF13 | red |
| RESF1 | red |
| RAPGEF2 | red |
| RAB27A | red |
| PELI1 | red |
| NUP153 | red |
| NUMB | red |
| NRBF2 | red |
| NAMPT | red |
| MARCKS | red |
| LRRK2 | red |
| IL18R1 | red |
| IFIT2 | red |
| GOLM2 | red |
| GCA | red |
| FOS | red |
| FAM174A | red |
| ERICH1 | red |
| ELOVL5 | red |
| DDX60L | red |
| CYB5R4 | red |
| CNEP1R1 | red |
| CMTM6 | red |
| CHMP1B | red |
| CD46 | red |
| ATP6V1A | red |
| ACAP2 | red |
| red-mon |  |
| S1PR1 | red |
| CDKN1B | red |
| ATM | red |
| midniteblue-neu |  |
| TSPAN31 | midnightblue |
| NENF | midnightblue |
| MRPL11 | midnightblue |
| MICOS13 | midnightblue |
| IFI27L1 | midnightblue |
| DENND4C | midnightblue |
| C19orf53 | midnightblue |
