# Supplementary material for: Contribution of FOS in neutrophils to venous thromboembolism via miR‐144 based on bioinformatic prediction and validation
Source: J Cell Mol Med. 2024 May 31;28(11):e18370. doi: 10.1111/jcmm.18370 (PMC11140234; doi:10.1111/jcmm.18370)
Supplement: Supplementary file 10 — Data S1. [file JCMM-28-e18370-s007.docx]

Supplementary file 1: The flow chart of the study.

Supplementary file 2: Sequences of primers used in this study.

Supplementary file 3: Detailed information of GSE19151, including P-values and fold changes.

Supplementary file 4: Detailed information of GSE48000, including P-values and fold changes.

Supplementary file 5: The KEGG pathway analysis and the enriched GO categories Biological Process, Cellular Component and Molecular Function for genes from Monocyte-red, M0 macrophage-green, neutrophil-red module, and neutrophil-midnight blue model.

Supplementary file 6: The genes in the module with module membership cutoff >0.8 and gene significance cutoff >0.5.

Supplementary file 7: CD46 expression levels of Whole-blood PCR and ELISA.

Supplementary file 8: FOS association with thrombus markers.

Supplementary file 9: The intersection genes of DEGs and FOS-regulated target genes.
